# Insulin receptor sensitization restores neocortical excitation/inhibition balance in a mouse model of autism

**DOI:** 10.1186/s13229-018-0196-6

**Published:** 2018-02-22

**Authors:** Fu-Sun Lo, Reha S. Erzurumlu

**Affiliations:** Department of Anatomy and Neurobiology, University of Maryland School of Medicine, 22 Penn Street HSFII-S259, Baltimore, MD 21201 USA

**Keywords:** Met receptor tyrosine kinase, Barrel cortex, Homeostatic plasticity, GABA_A_ receptors, Pioglitazone, Thalamocortical circuitry

## Abstract

**Background:**

Met receptor tyrosine kinase regulates neurogenesis, differentiation, migration, connectivity, and synaptic plasticity. The human *Met* gene has been identified as a prominent risk factor for autism spectrum disorder (ASD). Met gene-altered mice serve as useful models for mechanistic studies of ASD. Inactivation of *Met* in excitatory cortical neurons in mice (*Emx1*^*cre*^*/Met* ^*flox*^ mice) yields a phenotype in which significantly decreased GABA_A_ receptor-mediated inhibition shifts the excitation/inhibition (E/I) balance toward excitation in the somatosensory cortex. Further, unlike that seen in wild-type mice, insulin does not increase inhibition in the mutant cortex, suggesting that one of the consequences of kinase inactive *Met* gene could be desensitization of insulin receptors. To test this hypothesis, we investigated the effects of insulin receptor sensitizer, pioglitazone, on inhibition in the somatosensory thalamocortical circuitry.

**Methods:**

We used whole-cell patch clamp electrophysiology and analyzed excitatory and inhibitory responses of cortical layer IV excitatory cells following stimulation of their thalamic input in thalamocortical pathway intact brain slices. We applied insulin alone and insulin + a thiazolidinedione, pioglitazone (PIO), to test the effects of sensitizing insulin receptors on inhibitory responses mediated by GABA_A_ receptors in the somatosensory cortex of *Emx1*^*cre*^*/Met* ^*flox*^ mice.

**Results:**

In WT brain slices, application of insulin together with PIO did not enhance the effect of insulin alone. In contrast, PIO application induced a much larger inhibition than that of insulin alone in *Met*-defective cortex. Thus, insulin resistance of GABA_A_ receptor-mediated response in *Met* mutant mice may result from desensitized insulin receptors.

**Conclusions:**

Sporadic clinical studies reported improved behavioral symptoms in children with autism following PIO treatment. We show that PIO can aid in normalization of the E/I balance in the primary somatosensory cortex, a potential physiological mechanism underlying the positive effects of PIO treatment.

## Background

Numerous studies have associated *Met* gene as a of a functional risk allele in autism spectrum disorders (ASD) [1–3]. The Met receptor tyrosine kinase is a multifunctional receptor with diverse biological roles activating various intracellular signaling pathways. *Met* mutations in mice provide models to investigate cellular and molecular deficits in the nervous system. One such line, *Emx1*^*cre*^*/Met* ^*flox*^ mice (from here on referred to as *Met-Emx1* mouse), has kinase-inactive *Met* gene in excitatory neurons of the neocortex, hippocampus, and olfactory bulbs [4, 5]. For cortical studies, these mice may be considered as cortex-specific null mutation of *Met*. Previously, we reported that in thalamocortical brain slices from *Met-Emx1* mice the E/I ratio is biased toward excitation in the primary somatosensory cortex due to decreased GABA_A_ receptor-mediated inhibition [6]. Excitation-inhibition (E/I) balance in neural circuits is a type of homeostatic synaptic plasticity that is notably affected in various neurological and psychiatric conditions [7, 8].

Insulin receptors are abundant in the brain [9, 10], and insulin regulates GABAergic inhibition by increasing the density of GABA_A_ receptors, consequently augmenting inhibitory postsynaptic currents [11, 12]. In our study, we found that in wild-type (WT, C57BL/6) brain slices insulin enhances GABA_A_ receptor-mediated response. In contrast, insulin treatment had no effect on slices from *Emx1*^*cre*^*/Met* ^*flox*^ mice [6]. To determine whether sensitizing insulin receptors can enhance GABA_A_ receptor-mediated response, we tested pioglitazone (PIO) treatment. PIO is a widely used thiazolidinedione that acts as an insulin-sensitizer through activation of the peroxisome proliferator-activated receptor-γ [13]. There are two members of the class thiazolidinediones that have been used as insulin sensitizers in treatment of type 2 diabetes: pioglitazone (Actos) and rosiglitazone (Avandia). We have chosen to use PIO because it is the most widely used and rosiglitazone has yielded less-desirable effects. Furthermore, few clinical studies have reported improved symptoms in neurodevelopmental and neurogeriatric conditions following PIO treatment [14–20]. In the future, testing different members of this drug family under in vivo conditions could yield important insights. In WT thalamocortical slices, application of insulin plus PIO did not enhance the effect of insulin alone. However, this treatment induced a much larger inhibition than that of insulin alone in *Met-Emx1* mice. This finding indicates that the insulin resistance of GABA_A_ receptor-mediated response in *Met-Emx1* mice mainly results from desensitized insulin receptors.

## Methods

### Animals

The brain slices were derived from *Met-Emx1* mice of both sexes (*n* = 5). We obtained these mice through crossings of the floxed *Met* mouse (*Met-fx*) with the cerebral cortical and hippocampal specific *Emx1-cre* mouse. Our original sources and further details of the mouse lines are detailed in a previous paper [6], currently both lines are commercially available (*Met-fx* stock #016974, *Emx1-Cre*, stock #005628, Jackson Laboratory, Bar Harbor, ME). Control brain slices were from mice with *Met-fx* or *Emx1-cre* alleles alone or C57BL/6 (the background of the mutant line) mice (*n* = 5). There were no differences between the three types of control slices. For mouse use, we followed the National Institute of Health Guide for the Care and Use of Laboratory Animals (ISBN:13:978-0-309-15400-0, revised in 2011) and the UMB SOM Animal Use and Care Committee approved our protocol.

### Brain slice preparation

Two- to 4-week-old mice were euthanized and their brains removed into cold sucrose supplemented with artificial cerebrospinal fluid (ACSF, in mM: NaHCO_3_ 25, glucose 11, sucrose 234, KCl 2.5, NaHPO_4_ 1.25, MgSO_4_ 10). Next, we cut 350-μm-thick thalamocortical pathway intact brain slices using a vibratome (Campden 7000msz), at an angle of 50–55° from the mid-sagittal plane and 10° from the coronal plane [21, 22]. In such slices, the connections from the ventrobasal nucleus of the thalamus (VB) to the primary somatosensory, specifically the whisker representation zone, the barrel cortex, remain intact. We kept the slices in normal ACSF (in mM: NaCl 126, KCl 3.0, NaH_2_PO_4_ 1.25, MgSO_4_ 1.0, NaHCO_3_ 26, glucose 10, CaCl_2_ 2, l-ascorbic acid 1.3, pH = 7.4) for at least 1 h, at room temperature. We placed the slice containing the thalamocortical pathway in a submerged-type recording chamber (27 L, Warner Ins.), and continuously perfused (> 2 mL/min) with normal ACSF at room temperature. Under these conditions, intra-cortical circuits are inactivated, and the excitatory thalamocortical synapses on layer IV neurons can be isolated [23].

### Thalamocortical slice electrophysiology

We prepared borosilicate glass (WPI, K150F-4) whole-cell-patch micropipettes by pulling them in three stages with a P-87 horizontal puller (Sutter Instrument Co.). We backfilled the electrodes with a Cs-based intracellular solution (in mM: CsMeSO_3_, 115; NaCl, 10; KCl, 1, MgCl_2_, 4; CaCl_2_, 1; EGTA, 11; HEPES, 20; Na_2_-ATP, 3; Na_2_-GTP, 0.5, pH = 7.25, > 290 mOsm). The electrodes had a tip resistance of 5–9 MΩ. Spiny stellate and star pyramid cells are the excitatory neurons of layer IV barrel cortex. We patched these neurons to form whole-cell configuration. We passed depolarizing current pulses through the patch pipette in current clamp mode to identify the cells by their firing patterns [24–26]. For stimulation of the thalamocortical afferents, we used a concentric electrode (FHC, CBFP J50) either inserted in the VB, which can be visualized in slices or in the internal capsule (IC). We passed 0.33 Hz electrical pulses for 0.3 ms duration, 0.33 Hz to evoke postsynaptic responses in both current- and voltage-clamp modes. We acquired the by Axopatch 200B amplifier and an InstruTECH ITC-16 interface unit and stored on a Dell DM061 computer with PULSE (HEKA) software program.

### Isolation of GABA_A_ receptor- and AMPA receptor-mediated postsynaptic currents

At − 60 mV holding potential, stimulation of the VB elicits an early inward current (excitatory postsynaptic current, EPSC) followed by an outward current (inhibitory postsynaptic current, IPSC). As we move the holding potential toward more negative values, the outward current becomes smaller, and when this current disappears around − 70 mV holding potential, we define this as the GABA_A_ receptor reversal potential. In the absence of any NMDA receptor blockade, the remaining inward current is pure AMPA receptor-mediated EPSC. This inward current can be blocked by 10 μM NBQX, but not by 50 μM picrotoxin (PTX). Changing the holding potential toward more positive values decreased the amplitude of the inward current and it disappeared around 0 mV holding potential, which we defined as the reversal potential for glutamate receptors. The resulting outward current could be completely blocked by 50 μM PTX but not by 100 μM DL-APV; thus, it is the isolated GABA_A_ receptor-mediated IPSC. We calculated the ratio of AMPA/GABA (E/I) for each neuron by averaging 10 traces of EPSCs and IPSCs induced by maximal stimulation.

### GABA_A_ receptor-mediated spontaneous IPSCs (sIPSCs)

We recorded spontaneous IPSCs at 0 mV. We measured the averaged amplitude of sIPSCs using MiniAnalysis Software.

### Chemicals

Insulin (500 nM), pioglitazone (PIO, 10 μM), an insulin receptor sensitizer, NBQX (10 μM), an AMPA receptor antagonist, DL-APV (100 μM), a NMDA receptor antagonist, and PTX (50 μM), a GABA_A_ receptor antagonist were applied as needed. All chemicals are purchased from Sigma-Aldrich Co. (St. Louis, MO).

### Data analysis

We used Student’s *t* test to determine significance. All data are expressed by mean ± s.e.m. (standard error of the mean). Box and whisker plots were generated in R studio version 1.1.419.

## Results

### Functional insulin receptors in the barrel cortex of WT mice

**Fig. 1:**
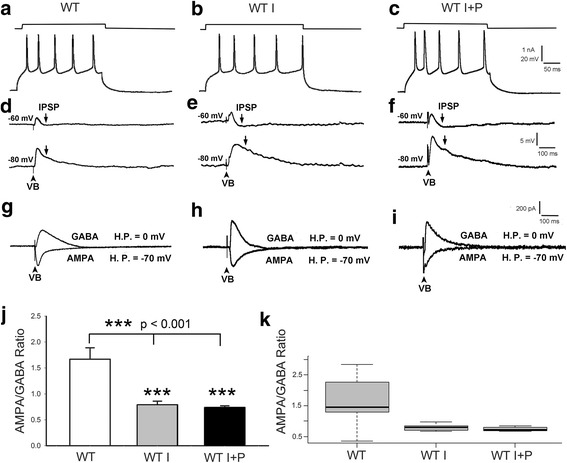
Insulin and pioglitazone treatment effects on the E/I ratio in WT brain slices. **a**–**c** Membrane depolarization characteristically leads to regular spiking of layer IV excitatory neurons. This characteristic is evident in control (WT) slices, and after application of insulin (WT I), or insulin + pioglitazone (WT I + P). **d** EPSP-IPSP sequence following VB stimulation. At − 60 mV, the IPSP does not hyperpolarize below − 60 mV (upper trace). The IPSP polarity reverses at − 80 mV, around GABA_A_ receptor reversal potential (lower trace). **e** 500 nM insulin application results in an increase of IPSP that hyperpolarized below base line at − 60 mV (upper trace). The IPSP is mediated by GABA_A_ receptor, because it reverses at − 80 mV (lower trace). **f** Addition of pioglitazone to insulin application does not lead to any notable change. **g**–**i** Representative records of GABA_A_ receptor- and AMPA receptor-mediated currents under control, insulin, and insulin + pioglitazone application conditions. *HP* holding potential. **j** The averaged AMPA/GABA (E/I) ratios under the three conditions. Note that insulin application significantly reduces the E/I ratio; addition of pioglitazone does not change insulin effects alone. **k** Box and whisker plots showing the population distribution

Previously, we investigated the thalamocortical synaptic transmission in the barrel cortex of various lines of transgenic and WT mice [6, 27, 28]. In complete agreement with those studies, layer IV excitatory neurons show adapting discharges (regular spikes, RS) upon membrane depolarization (Fig. 1a–c). Stimulation of VB induces an excitatory postsynaptic potential-inhibitory postsynaptic potential (EPSP-IPSP) sequence. At membrane potential of − 60 mV, the IPSP just curtails the EPSP but does not hyperpolarize below − 60 mV (Fig. 1d, upper trace). The IPSP is reversed in polarity at − 80 mV, suggesting that it is mediated by GABA_A_ receptor with a reversal potential around − 70 mV (Fig. 1d, lower trace).

In WT thalamocortical slices, application of 500 nM insulin resulted in an increase of IPSP that hyperpolarized below base line at − 60 mV (Fig. 1e, WT I, upper trace). The IPSP was also mediated by GABA_A_ receptor, because it reversed at − 80 mV (Fig. 1e, lower trace). In order to quantify the changes in E/I ratio, we voltage-clamped each cell to the reversal potential of GABA_A_ receptors (around − 70 mV) and AMPA receptors (around 0 mV) so that we could record GABA_A_ receptor- and AMPA receptor-mediated currents (1; 25). Figure 1g, h are representative records. The averaged AMPA/GABA (E/I) ratio for control WT mice was 1.67 ± 0.22 (*n* = 11, Fig. 1j, k), while insulin significantly (*p* < 0.001) reduced the ratio to 0.79 ± 0.07 (*n* = 7, Fig. 1j, k). To test whether the effect of insulin can be further enhanced by insulin receptor sensitizer PIO, we applied both 500 nM insulin and 10 μM PIO (I+P) to the in vitro preparation. The results were largely similar to those obtained with insulin application alone (Fig. 1c, f, i, j, k). The averaged AMPA/GABA ratio was 0.74 ± 0.03 (*n* = 6, Fig. 1j, k), which is about the same as insulin alone (*p* > 0.53). Thus, PIO did not increase the effect of insulin on GABA_A_ receptor-mediated response in WT slices, suggesting that insulin receptors are saturated by 500 nM insulin.

**Fig. 2:**
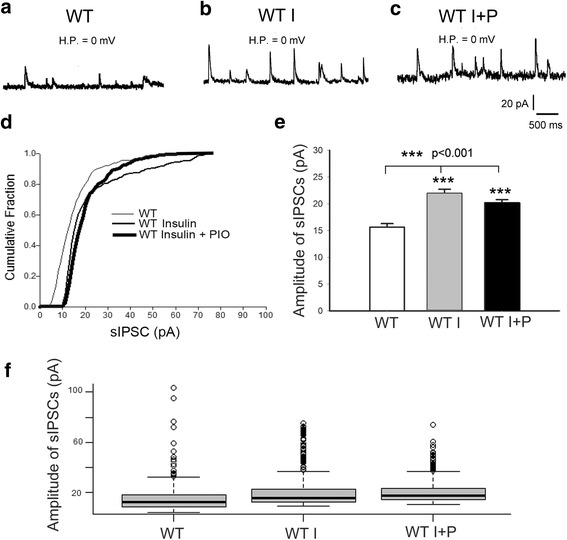
Insulin-induced increased inhibition. **a**–**c** Example records of GABA_A_ receptor-mediated sIPSCs. *HP* holding potential. **d** Cumulative fraction curves of the sIPSC amplitudes show that the amplitude distribution for insulin (WT I) and insulin + pioglitazone (WT I + P) are shifted to the right (larger amplitude), indicating that both conditions increase the amplitude of sIPSCs compared to no drug controls. **e** The average amplitude of sIPSCs is higher with either drug application condition compared to the controls, but not different between the two drug application conditions. **f **Box and whisker plots showing the population distribution

To investigate the mechanism underlying insulin-induced increased inhibition, we recorded GABA_A_ receptor-mediated spontaneous IPSCs (sIPSCs) as shown in Fig. 2a–c. The cumulative fraction curves of the amplitude of sIPSCs (Fig. 2d) show that the amplitude distribution for WT I (medium thickness line) and WT I + P (thick line) is shifted to the right (larger amplitude) than that of no insulin controls (WT, thin line). This indicated that insulin (I) and insulin + PIO (I + P) increased the amplitude of sIPSCs. The difference between WT I and WT I + P curves resulted from the difference in amplitude distribution. WT I + P group had less (12%) larger (> 30 pA) sIPSCs and WT I group had (19%) larger sIPSCs. However, the averaged amplitude of sIPSCs for WT I and WT I + P (Fig. 2e, f) showed no significant difference (*p* > 0.08), because the averaged amplitude of sIPSCs of WT I was 22.00 ± 0.76 (*n* = 434) pA, while that for WT I + P was 20.22 ± 0.56 (*n* = 319). Both of them were significantly (*p* > 0.001) larger than the WT group (15.64 ± 0.68 pA, *n* = 320).

### Insulin receptors are desensitized in the barrel cortex of *Met-Emx1* mice

**Fig. 3:**
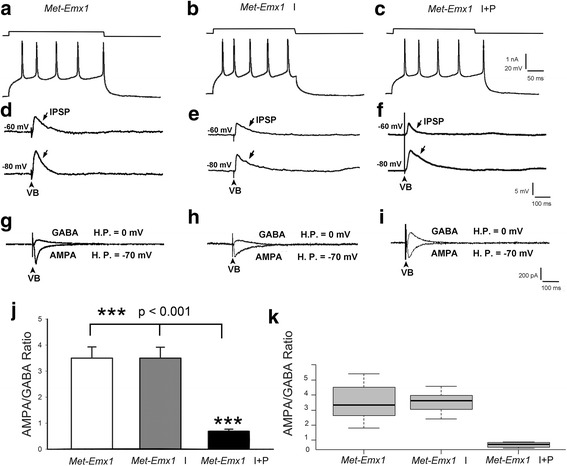
Insulin and pioglitazone treatment effects on the E/I ratio in *Met-EMx1* brain slices. **a**–**c** Regular spiking of layer IV excitatory neurons in control (*Met-EMx1*) slices, and after application of insulin (*Met-EMx1* I), or insulin + pioglitazone (*Met-EMx1* I + P). **d**, **g** In mutant brain slices, stimulation of VB induced mainly an EPSP without a clear cut IPSP. Isolated AMPA receptor- and GABA_A_ receptor-mediated currents reveal small IPSC. **e**, **h** Application of insulin (*Met-EMx1* I) did not change the magnitude of GABA_A_ receptor-mediated response. **f**, **i** Addition of pioglitazone to insulin application dramatically increased GABA_A_ receptor-mediated inhibition. **j** The averaged AMPA/GABA (E/I) ratio is similar between no drug treatment and insulin alone treatment conditions, but application of pioglitazone along with insulin dramatically changed this ratio. **k** Box and whisker plots showing the population distribution

Layer IV of barrel cortex excitatory neurons of *Met-EMx1* mice also show regular spiking upon membrane depolarization (Fig. 3a–c). Stimulation of VB induced mainly an EPSP without a clear cut IPSP in *Me-Emx1* mice (Fig. 3d). Isolated AMPA receptor- and GABA_A_ receptor-mediated currents revealed a relatively smaller IPSC (Fig. 3g). The averaged AMPA/GABA ratio for *Met-Emx1* mice was 3.53 ± 0.43 (*n* = 8, Fig. 3j, k), which is significantly (*p* < 0.001) larger than that of the WT mice (1.67 ± 0.22). In contrast to the WT slices, application of insulin (*Met-Emx1* I) did not change the magnitude of GABA_A_ receptor-mediated response (Fig. 3e, h, j, k). The averaged AMPA/GABA ratio was 3.53 ± 0.42 (*n* = 5) that was just the same as without insulin (*p* > 0.99). The failure of insulin effect on inhibition may result from desensitized insulin receptors; thus, we applied PIO together with insulin (*Met* I + P). Representative records (Fig. 3f, i) demonstrated that addition of PIO increased GABA_A_ receptor-mediated inhibition so that the averaged AMPA/GABA ratio was 0.69 ± 0.08 (*n* = 5), which is significantly (*p* < 0.001) smaller than previous groups (Fig. 3j, k).

**Fig. 4:**
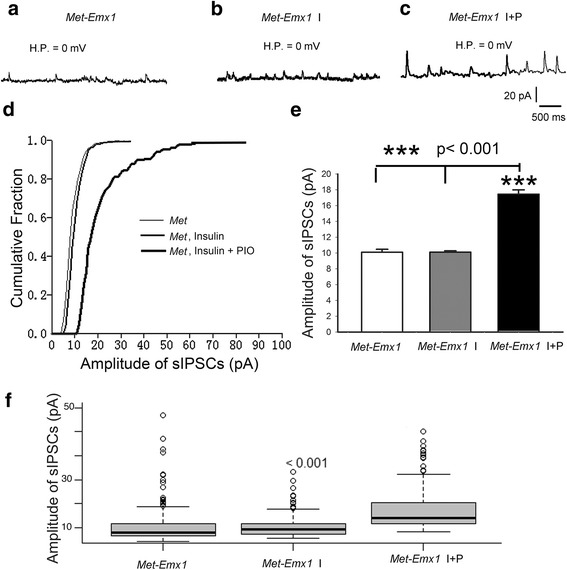
Insulin sensitization-increased inhibition in *Met-EMx1* brain slices**. a**–**c** Example records of GABA_A_ receptor-mediated sIPSCs in *Met-EMx1* cortical neurons without drug application, and with insulin alone (I) or insulin + pioglitazone (I + P) applications. **d** Cumulative fraction curves of the sIPSC amplitudes show a significant shift of the amplitude distribution for insulin + pioglitazone condition compared to no drug treatment (*Met-EMx1*) or insulin treatment (*Met-EMx1* I) alone. **e** The average amplitude of sIPSCs is much higher following insulin receptor sensitization with pioglitazone. **f **Box and whisker plots showing the population distribution

We also recorded GABA_A_ receptor-mediated sIPSCs in *Met-Emx1* mice. Representative records (Fig. 4a–c) showed that the amplitudes of sIPSCs increased only during application of insulin plus PIO (Fig. 4c). The cumulative fraction curves for *Met-Emx1* and *Met-Emx1* I were about the same (Fig. 4d, thin and medium lines). However, the curve for *Met-Emx1* I + P was shifted to the right, the larger amplitude area (Fig. 4d, thick line). The averaged amplitude of sIPSCs for *Met-Emx1* mice was 10.10 ± 0.37 pA (*n* = 295, Fig. 4e, f) that was significantly (*p* < 0.001) smaller than that of WT mice (15.64 ± 0.68 pA). However, application of insulin (*Met* I) did not change (*p* > 0.97) the averaged amplitude of sIPSCs (10.11 ± 0.17 pA, *n* = 470, Fig. 4e, f), suggesting insulin resistance of GABA_A_ receptor-mediated response. Additional PIO together with insulin remarkably (*p* < 0.001) increased the amplitude of sIPSCs to 17.44 ± 0.55 pA (*n* = 235, Fig. 4e, f) that was still smaller (*p* < 0.001) than WT I + P (20.22 ± 0.56 pA). These suggest that insulin receptors are desensitized in *Met-Emx1* mice. The density of insulin receptors in *Met-Emx1* mice may be lower than that in WT mice.

## Discussion

E/I balance in neural circuits is an essential component of homeostatic synaptic plasticity, which is thought to restrain cortical network activity to operate at optimal levels by weakening synaptic efficacy after heightened activity and increasing it after low levels of activation [29]. A disturbed E/I balance, particularly in neocortical circuits, has been proposed and observed in ASD individuals and in animal models [7, 30, 31].

In neurodevelopmental disorders, failure in neuronal differentiation [32], defects in neurotransmitter release, and their postsynaptic receptors [33–35] have been linked to altered E/I balance. In neocortical circuits, interactions between Gamma-aminobutyric acidergic (GABAergic) interneurons and glutamatergic pyramidal neurons that form the corticocortical, callosal, and subcortical connections have been targeted by numerous studies seeking to unveil the circuit defects in disorders with cognitive symptoms. GABAergic neurons are a small population of neocortical residents, but they control inhibition of the most populous excitatory cortical neurons. Defects in differentiation of GABAergic interneurons, GABAergic synaptic transmission, and postsynaptic receptors have been the usual suspects in altered excitability and homeostatic plasticity of neural circuits.

Met receptor tyrosine kinase is a cell surface receptor activated by hepatocyte growth factor (HGF). For obvious reasons, Met and HGF signaling have been extensively studied in the liver. Met is structurally related to the insulin receptor tyrosine kinase and Met signaling is essential in regulation of insulin response by hepatocytes [36]. In fact, a potential therapeutic role for HGF treatment for insulin resistance in type 2 diabetes has been suggested [36]. A more recent study on cell cocultures and in knockout mice brings conclusive evidence for Met regulation of insulin sensitivity [37]. While both Met and HGF are expressed in a spatiotemporal specificity in the brain (reviewed in [1]), there are no studies examining any link between Met signaling and insulin sensitivity in neocortical excitatory neurons and how lack of it might affect GABAergic synaptic transmission.

In the primary somatosensory cortex of mice, with inactive autism-associated Met receptor tyrosine kinase, we found that increased excitation is due to decreased postsynaptic inhibition mediated via the GABA_A_ receptors [6]. Altered GABA_A_ receptor function can directly affect the E/I balance. In transgenic mice lacking the β3 subunit of the GABA_A_ receptor (Gabrb3), seizure susceptibility, hypersensitivity, hyperactivity, learning, and memory deficits have been reported [38, 39]. In fragile X mouse models too, decreased GABA_A_ receptor expression has been observed [40–42]. A potential strategy to ameliorate decreased GABA_A_ receptor function is to increase receptor sensitivity. In cultured hippocampal neuron, application of insulin increased GABA_A_ receptor-mediated response [43, 44]. We confirmed this response in thalamocortical slices taken from WT mice [6]. However, in thalamocortical slices from *Met-Emx1* mice, insulin application did not change GABA_A_ receptor-mediated response, suggesting that there might be insulin resistance in neocortical neurons that lack Met function [6]. In the present study, we show that insulin resistance of GABA_A_ receptor-mediated response in *Met-Emx1* mice can be altered by insulin sensitization. We find that application of PIO, a common insulin sensitizer used in diabetes therapy, can significantly alter the GABA_A_ receptor response and restore E/I balance to levels of normalcy. At the present time, we do not know how Met deficiency in excitatory cortical neurons alters receptor desensitization or the intracellular signaling pathways involving insulin receptors. Further studies are needed to investigate these mechanisms in a cell type-specific manner to determine whether Met signaling through insulin receptors occurs in similar ways between the neocortical excitatory neurons and hepatocytes.

## Conclusions

Excitation/inhibition balance is altered in favor of excitation in the primary somatosensory cortex of mice with inactive autism-associated Met receptor tyrosine kinase. Altered E/I balance, in favor of excitation, in the somatosensory cortex of mice with inactive autism-associated Met receptor tyrosine kinase may relate to somatic sensory hypersensitivity of children with ASD to shoes and clothing. We applied insulin alone and insulin + pioglitazone (PIO) to test the effects of sensitizing insulin receptors on inhibitory responses mediated by GABA_A_ receptors in the thalamocortical circuitry of *Emx1*^*cre*^*/Met* ^*flox*^ mice. We show that PIO can aid in normalization of the E/I balance in the primary somatosensory cortex, a potential physiological mechanism underlying the positive effects of PIO treatment in ASD patients. Our results shed light to the underlying mechanisms and suggest that decreased GABA_A_ receptor activity is the major culprit in scaling E/I balance to more excitation and that insulin sensitization can help in readjusting the balance.

## Abbreviations

ACSF: Artificial cerebrospinal fluid
AMPA: α-Amino-3-hydroxy-5-methyl-4-isoxazolepropionic acid
ASD: Autism spectrum disorder
C57BL/6: Most widely used inbred mouse strain
DL-APV: DL-2-Amino-5-phosphonopentanoic acid
E/I: Excitation/inhibition
Emx1: Empty spiracles homeobox
EPSC: Excitatory postsynaptic current
GABA_A_: Ionotropic receptor and γ-aminobutyric acid-gated ion channel
Gabrb3: β3 subunit of the GABA_A_ receptor
HGF: Hepatocyte growth factor
I + P: Insulin + pioglitazone
I: Insulin
IPSC: Inhibitory postsynaptic current
Met: Tyrosine-protein kinase Met or hepatocyte growth factor receptor
NBQX: 2,3-Dihydroxy-6-nitro-7-sulfamoyl-benzo(F) quinoxaline, potent AMPA receptor antagonist
NMDA: *N*-methyl-d-aspartate
PIO: Pioglitazone
PTX: Picrotoxin
sIPSC: Spontaneous inhibitory synaptic current
VB: Ventrobasal nucleus of thalamus
WT: Wild type (B6 mice)
